# Body Composition and Serum Total Calcium Were Associated With Blood Pressure Among Children and Adolescents Aged 7–18 in China: A Cross-Sectional Study

**DOI:** 10.3389/fped.2019.00411

**Published:** 2019-10-09

**Authors:** Huijing He, Li Pan, Jianwei Du, Feng Liu, Yuming Jin, Jingang Ma, Li Wang, Pengben Jia, Zhiping Hu, Guangliang Shan

**Affiliations:** ^1^Department of Epidemiology and Statistics, Institute of Basic Medical Sciences, Chinese Academy of Medical Sciences & School of Basic Medicine, Peking Union Medical College, Beijing, China; ^2^Hainan Provincial Center for Disease Control and Prevention, Haikou, China; ^3^Shaanxi Provincial Center for Disease Control and Prevention, Xi'an, China

**Keywords:** blood pressure, children, adolescent, body mass index, serum total calcium, risk factor

## Abstract

**Background:** This study aimed to assess the prevalence of childhood pre-hypertension and hypertension (elevated blood pressure, EBP) and explore their risk factors, especially the role of body composition and serum total calcium on EBP.

**Methods:** From Nov 2013 to Jul 2014, a cross-sectional study was conducted in Hainan and Shaanxi Provinces in China. Two thousand two hundred eighty-three children and adolescents aged 7–18 underwent anthropometric and blood pressure measurements. Fasting blood was collected and serum total calcium was tested. Blood pressure standards from the updated Fourth Report on the Diagnosis, Evaluation, and Treatment of High Blood Pressure in Children and Adolescents were used to classify BP groups based on age, sex and stature.

**Results:** The overall prevalence of pre-hypertension and hypertension were 8.36 and 7.06%, respectively. Boys, older age, living in Hainan Province, excess adiposity and higher level of serum total calcium were found to be associated with EBP. Lean subjects had an average 3.87 mmHg decrease in systolic blood pressure (SBP) than normal weight groups. In contrary, overweight/obesity had increased SBP (3.69 mmHg) and diastolic blood pressure (DBP) (2.58 mmHg) than the normal weight group. Participants with high-level serum total calcium had a 1.60 mmHg increased SBP and 1.77 mmHg increased DBP than the low-level group. Compared with normal weight individuals, lean subjects appeared to have decreased odds of EBP (OR = 0.65, 95% CI: 0.43–0.98) but overweight/obese individuals were more likely to have EBP (OR = 2.67, 95% CI: 1.68–4.25). High-level of serum total calcium was associated with increased odds of EBP, the OR (95% CI) was 1.51 (1.17–1.94). The restrict cubic spline presented a linear relationship between serum total calcium and OR of EBP (*P* < 0.001).

**Conclusions:** Body composition and serum total calcium were positively associated with blood pressure in children and adolescents. Serum calcium could be considered as an untraditional risk factors for high blood pressure screening along with other body composition indexes in clinical settings.

## Introduction

Previous evidence emphasized that adult cardiovascular disease (CVD) begins in child (1, 2). As the most populous country in the world, China has a high hypertension burden that almost half population has elevated blood pressure (EBP) (3). Pediatric elevated blood pressure (EBP) tends to track into adulthood (4) and could result in a series of early target organ damages in childhood (5). Prevention and control of EBP from early age is of high health priority in China. The prevalence of EBP among children and adolescents has been increasing in line with the increasing prevalence of overweight and obesity among the youths (6–8). Based on the China National Nutrition and Health Survey, the prevalence of overweight in children and adolescents aged 7–18 increased from 4.1 in 2000 to 8.1% in 2010 (9, 10).

Since pediatric hypertension is usually asymptomatic and can easily be missed by clinicians (11), understanding its risk factors among children, and adolescents may help health professionals to identify high risk population. Excess adiposity was considered the most important risk factor for EBP in children and juveniles (12, 13). A cross-sectional survey in middle China showed that there was a dose-response relationship between obesity and childhood EBP (14). Another study among Chinese adolescents aged 12–17 also supported that overweight/obesity was independently associated with EBP (15).

Of note, some biomarkers have been found in identifying high risk among the young (16, 17), which are useful in clinical settings. As one of these biomarkers, serum calcium was noted as it played an important pathophysiologic role in cardiovascular and kidney function (18, 19). The association between serum calcium and hypertension has been studied in adults with different races or ethnicities (17, 18, 20–22). Data derived from the Third National Health and Nutrition Examination Survey in the United States indicated that serum total calcium was positively associated with hypertension in the US adults (18). However, this association is still unclear in children and adolescents.

In 2013, a cross-sectional survey was designed to understand associated factors of childhood CVD risk factors, including EBP. Serum total calcium and other demographic, anthropometric, and biochemical information was collected. Serum total calcium is the total sum of three forms, ionized, protein-bound, and soluble from complexed with anions such as bicarbonate and phosphate (18). The objective of this study is to assess the prevalence of EBP, and effect of serum total calcium and excess adiposity on EBP in children and adolescents aged 7–18 years in China.

## Methods and Materials

### Study Participants

**Figure 1:**
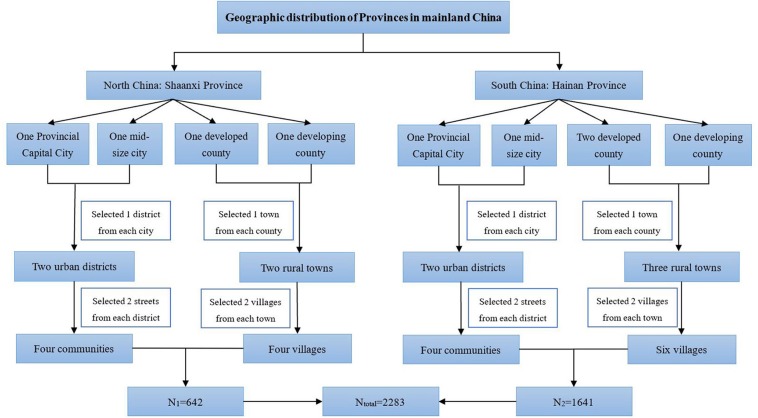
Flow chart of the sampling method. A multi-stage stratified cluster sampling method was used in 2013 to enroll children and adolescents aged 7–18 years of China (23).

From Nov 2013 to Jul 2014, 2,283 children and adolescents living in Hainan (South China) and Shaanxi (Northwest China) Provinces participated in the study. A multi-stage stratified cluster sampling method was used to select subjects (sampling details were provided in Figure 1: the flow chart of the sampling method) (23). Children and adolescents aged 7–18 and their parents who had local residency for at least 1 year were eligible to participate. Any current intake of medication for hypertension or other chronic diseases was considered as the exclusion criteria. The sample size calculation was based on an estimated prevalence of childhood EBP of 10% (p). To reach a significance level (alpha) of 0.05 and error tolerance 0.15 × p, the estimated minimum sample size was 1,537. We added an additional 20% to the minimum sample size factoring in possible non-compliance rate and targeted 1,845 subjects, with the same sex and age stratification. Finally, 2,283 children and adolescents participated in the survey. Ethical approval was obtained from the Bioethical Committee of Institute of Basic Medical Sciences, Chinese Academy of Medical Sciences. All participants provided parental written informed consent before the survey.

### Measurements

The parents of children and adolescents were investigated using an interviewer-administered questionnaire at the survey settings. Information on demographic characteristics (age, sex, ethnic group, urban or rural residency) and physical exercise of the youths was collected.

Stature was measured to 0.1 cm using a fixed stadiometer. Weight and body fat percentage (%BF) were measured after a fasting period of at least 8 h, barefooted, and with light clothes using body composition analyzer (TANITA BC-420, Japan) with decimal accuracy. BMI was calculated as weight in kilograms divided by the square of stature in meters (kg/m^2^).

Systolic blood pressure (SBP) and diastolic blood pressure (DBP) were measured on the right arm using a digital blood pressure measuring device (Omron HEM-907, Japan) with children customized cuff size after one subject rested for at least 5 min in a sitting position. Three readings were measured with 1-min interval and the average value was recorded. We collected overnight fasting venous blood samples for the measurements of serum total calcium and serum lipids by Chemistry Analyzer (ROCHE Cobas8000C701, USA) at the General Hospital of Chinese People's Liberation Army (PLA).

### Definitions

Blood pressure was classified into normal BP, pre-hypertension and hypertension based on the updated guidelines of the *Fourth Report on the Diagnosis, Evaluation, and Treatment of High Blood Pressure in Children and Adolescents* (24). For children younger than 13 years, normal blood pressure (BP) was defined as SBP and DBP no more than 90th percentile for age, sex, and stature specific reference. Hypertension (HTN) was defined as SBP and/or DBP greater than the 95th percentile. SBP and/or DBP greater than 90th percentile but less than the 95th percentile, or SBP greater than 120 mmHg or DBP greater than 80 mmHg defined pre-hypertension (pre-HTN). For those aged 13 or above, normal BP was defined as SBP < 120 and DBP < 80 mmHg; pre-HTN was defined as 120/<80–129/<80 mmHg; HTN was defined as SBP > 130 or DBP > 80 mmHg. EBP in the current study was defined as either pre-HTN or HTN.

Thinness, normal weight, overweight, and obesity was defined according to criteria of world health organization (WHO) for children and adolescents 5–19 years old (25): thinness was BMI-for-age lower than two standard deviations below the WHO Growth Reference median; overweight was BMI-for-age >1 standard deviation and obesity was > 2 standard deviations. Physical exercise was categorized as regular (<3 times per week, 20 min per time.), occasionally (one or two times per week) and never (<3 times per month). Serum total calcium level of each subject was defined as either low or high based on its comparison with the median value, which was 2.47 mmol/L, of the whole population.

Dyslipidemia was defined according to the National Heart, Lung, and Blood Institute (NHLBI) cholesterol screening guidelines and cut points backed by the American Academy of Pediatrics (26).

### Statistical Analyses

Shapiro-Wilk tests were used to verify the normality of the distribution of continuous variables. Continuous data were shown as mean and standard deviation. Categorical data were described as numbers and percentages. For continuous variables, Student's *t* test or Mann-Whitney *U* test (when the data were not normally distributed) was used to perform two-group comparisons. Logistic regression models were used to calculate the multi-variable adjusted prevalence of pre-HTN and HTN (27). Two-way analysis of covariance was performed using general linear regression models (GLMs) to identify the adjusted associations between covariates and blood pressure (SBP and DBP). Multivariable logistic models were used to investigate the associators of EBP. In this step, there were three models based on covariates considered in the regressions: Model 1 only considered sex and age; Model 2 further considered BMI based on Model 1; and Model 3 additionally considered other covariates such as urbanization, ethnicity, serum calcium level and physical exercises based on Model 2. We further did sub-group analysis within age group to explore the association between BMI and serum total calcium with EBP. We examined the possible non-linear association between body mass index, serum total calcium, and EBP using restricted cubic splines; tests for non-linearity used the likelihood ratio test, comparing the model with only the linear term to the model with the linear and the cubic spline terms (28). A *P*-value <0.05 (two-tailed) was considered statistically significant. Because there were limited number of obesity in both boys and girls, obesity was combined with overweight as one category, presented as overweight/obesity in the results section.

In order to compare the results with other Chinese studies which used different definition of EBP, we further calculated pre-HTN and HTN prevalence using the updated blood pressure reference for Chinese children and adolescents released in 2017 (29).

The statistical analyses were performed using SAS version 9.4 for windows (SAS Institute Inc, Cary, NC, USA).

## Results

### Basic Characteristics of Participants

A total of 2,283 children and adolescents (1,032 boys and 1,251 girls) participated in the survey. After excluding subjects with missing values on SBP or DBP, 2,237 participants were included in the final analyses. The population's general characteristics and the prevalence of pre-HTN and HTN were presented in Table 1, stratified by sex.

**Table 1:** Baseline characteristics of participants [(*n*, %) for categorical data and (mean, SD) for continuous data].

	**Boys (*****n*** **=** **1,006)**					**Girls (*****n*** **=** **1,231)**					**Total (*****n*** **=** **2,237)**				
		**Pre-hypertension**		**Hypertension**			**Pre-hypertension**		**Hypertension**			**Pre-hypertension**		**Hypertension**	
**Age group**	***N***	***n***	**%**	***n***	**%**	***N***	***n***	**%**	***n***	**%**	***N***	***n***	**%**	***n***	**%**
7–8	72	1	1.39	1	1.39	90	5	5.56	1	1.11	162	6	3.70	2	1.23
9–10	207	12	5.80	4	1.93	250	9	3.60	10	4.00	457	21	4.60	14	3.06
11–12	223	19	8.52	8	3.59	215	13	6.05	9	4.19	438	32	7.31	17	3.88
13–14	176	21	10.23	11	6.25	142	11	7.75	14	9.86	318	29	9.12	25	7.86
15–16	153	45	19.61	6	13.73	235	3	1.28	18	7.66	388	33	8.51	39	10.05
17–18	175	76	26.86	7	20.57	299	19	6.35	25	8.36	474	66	13.92	61	12.87
**REGIONS**															
Hainan (South China)	681	99	14.54	61	8.96	928	56	6.03	67	7.22	1,609	155	9.63	128	7.96
Shaanxi (North China)	325	28	8.62	20	6.15	303	4	1.32	10	3.30	628	32	5.10	30	4.78
**ETHNICITY**															
Han	658	91	13.83	54	8.21	708	33	4.66	36	5.08	1,366	124	9.08	90	6.59
Li	284	28	9.86	23	8.10	437	18	4.12	36	8.24	721	46	6.38	59	8.18
Others	64	8	12.50	4	6.25	86	9	10.47	5	5.81	150	17	11.33	9	6.00
**RESIDENTIAL AREAS**															
Urban	500	85	17.00	44	8.80	694	39	5.62	48	6.92	1,194	124	10.39	92	7.71
Rural	506	42	8.30	37	7.31	537	21	3.91	29	5.40	1,043	63	6.04	66	6.33
**FAMILY HISTORY OF HTN**															
No	701	96	13.69	57	8.13	818	39	4.77	48	5.87	1,519	135	8.89	105	6.91
Yes	305	31	10.16	24	7.87	411	21	5.11	28	6.81	716	52	7.26	52	7.26
**BMI CATEGORY**															
Thinness	143	10	6.99	9	6.29	136	3	2.21	11	8.09	279	13	4.66	20	7.17
Normal weight	763	100	13.11	62	8.13	1038	51	4.91	63	6.07	1,801	151	8.38	125	6.94
Overweight/obesity	100	17	17.00	10	10.00	57	6	10.53	3	5.26	157	23	14.65	13	8.28
**BODY FAT PERCENTAGE**															
Q1	244	16	6.56	14	5.74	302	11	3.64	13	4.30	546	27	4.95	27	4.95
Q2	256	28	10.94	16	6.25	312	16	5.13	15	4.81	568	44	7.75	31	5.46
Q3	253	41	16.21	27	10.67	305	15	4.92	23	7.54	558	56	10.04	50	8.96
Q4	253	42	16.60	24	9.49	311	18	5.79	25	8.04	564	60	10.64	49	8.69
**SERUM CALCIUM**															
Low	449	42	9.35	27	6.01	631	23	3.65	32	5.07	1,080	65	6.02	59	5.46
High	542	82	15.13	52	9.59	583	37	6.35	42	7.20	1,125	119	10.58	94	8.36
**PHYSICAL EXERCISE**															
Low	71	11	15.49	4	5.63	160	10	6.25	7	4.38	231	21	9.09	11	4.76
Moderate	579	84	14.51	48	8.29	726	36	4.96	52	7.16	1,305	120	9.20	100	7.66
High	353	32	9.07	29	8.22	344	14	4.07	18	5.23	697	46	6.60	47	6.74
**DYSLIPIDEMIA**															
No	799	103	12.89	60	7.51	952	49	5.15	48	5.04	1,751	152	8.68	108	6.17
Yes	192	21	10.94	19	9.90	262	11	4.20	26	9.92	454	32	7.05	45	9.91

*HTN, hypertension; BMI, body mass index; Q1–Q4, the first to forth quartile of body fat percentage. The classification of low or high in serum calcium level was based on its median value in the overall participants, which was 2.47 mmol/L*.

### Prevalence of Pre-HTN and HTN

In the overall population, boys and girls, the prevalence of pre-HTN were 8.36, 12.62, and 4.87%, respectively; and the HTN prevalence were 7.06, 8.05, and 6.26%, respectively. The age adjusted prevalence of pre-HTN and HTN in boys were 13.20% (95% CI: 11.10–15.60%) and 7.11% (95% CI: 5.65–8.91%), respectively, and in girls 4.29% (95% CI: 3.27–5.60%) and 4.90% (95% CI: 3.82–6.28%), respectively. Compared with girls, boys had higher prevalence of pre-HTN (*P* < 0.001) and HTN (*P* = 0.019).

The prevalence of pre-HTN and HTN were much higher in participants from Hainan province (9.63 and 7.96%) than those from Shaanxi (5.10 and 4.78%). After the adjustment of sex, age, urbanization and BMI status, the prevalence of pre-HTN (9.91%, 95% CI: 7.60–12.80%) and HTN (8.65%, 95% CI: 6.62–11.2%) in Hainan were still higher than in Shaanxi Province (pre-HTN: 3.68%, 95% CI: 2.41–5.56%; HTN: 3.82%, 95% CI: 2.49–.82%; *P* < 0.001).

The prevalence of HTN had increased with age in both sexes (*P* for trend < 0.001 in boys, and 0.001 in girls). However, when it came to pre-HTN prevalence, this trend was only observed in boys (*P* for trend <0.001, and *P* = 0.706 in girls).

### Factors That Influencing BP

After adjusting for age, sex and study sites, overweight/obese subjects had the highest prevalence of pre-HTN (15.20% with 95% CI of 10.00–22.50%) than normal weight (5.55% with 95% CI of 4.37–7.03%, *P* < 0.001) and lean (2.51% with 95% CI of 1.38–4.53%, *P* < 0.001) participants. For HTN, the prevalence among lean and normal weight youths were 4.90% (95% CI: 3.03–7.82%) and 4.68% (95% CI: 3.65–5.98%), respectively. Overweight/obese subjects also had the highest prevalence of HTN (9.23%, 95% CI: 5.36–15.4%) than the other two groups.

Results of GLMs were presented in Table 2. Boys, older age, Hainan Province, excess adiposity (measured by BMI and %BF) and higher serum total calcium level were associated with EBP. Boys had an average 4.75 mmHg higher SBP than girls, and for every 2 years increase in age, there were 3.07 mmHg increased SBP and 1.87 mmHg increased DBP, respectively. Compared with subjects living in Shaanxi Province, Hainan children and adolescents had 3.06 mmHg increased SBP and 1.47 mmHg increased DBP. Lean subjects had 3.85 mmHg decreased SBP than normal weight subjects. In contrary, overweight/obese children, and adolescents had increased SBP and DBP. Serum total calcium was associated with blood pressure. Compared with the low-level serum calcium group, the high-level group had 1.57 mmHg increased SBP and 1.74 mmHg increased DBP, respectively.

**Table 2:** The associations between participants characteristics and blood pressure in children and adolescents aged 7–18 years in China, 2014.

	**SBP**								**DBP**							
	**Before adjustment**				**After adjustment**				**Before adjustment**				**After adjustment**			
	***β***	**95%** ***CI ofβ***		***P***	**β**	**95%** ***CI ofβ***		***P***	**β**	**95%** ***CI ofβ***		***P***	**β**	**95%** ***CI ofβ***		***P***
Boys (ref = girls)	4.746	3.813	5.678	<0.001	5.621	4.790	6.452	<0.001	−0.756	−1.448	−0.063	0.032	−0.249	−0.902	0.404	0.454
Age (every two years increase)	3.038	2.775	3.302	<0.001	3.066	2.793	3.338	<0.001	1.846	1.648	2.045	<0.001	1.868	1.654	2.082	<0.001
Hainan (ref = Shaanxi)	2.188	1.137	3.239	<0.001	3.062	1.930	4.193	<0.001	1.960	1.197	2.723	<0.001	1.471	0.582	2.361	0.001
Li ethnicity (ref = Han)	−1.275	−2.306	−0.245	0.015	−0.613	−1.656	0.430	0.250	0.309	−0.440	1.059	0.418	0.423	−0.397	1.243	0.312
Urban (ref = rural)	4.812	3.883	5.742	<0.001	0.810	−0.119	1.738	0.088	2.716	2.034	3.398	<0.001	0.067	−0.663	0.797	0.858
Family history of HTN (ref = negative)	−0.506	−1.522	0.510	0.329	0.262	−0.620	1.144	0.560	0.319	−0.420	1.058	0.397	0.575	−0.118	1.268	0.104
Thinness (ref = normal weight)	−4.233	−5.663	−2.803	<0.001	−3.850	−5.098	−2.602	<0.001	−1.030	−2.078	0.018	0.054	−0.487	−1.468	0.494	0.331
Overweight/obesity (ref = normal weight)	1.764	−0.085	3.613	0.062	3.729	2.056	5.403	<0.001	0.623	−0.733	1.979	0.368	2.472	1.157	3.787	<0.001
%BF (every 5% increase) *	0.633	0.358	0.908	<0.001	0.755	0.455	1.055	<0.001	0.902	0.704	1.099	<0.001	0.484	0.250	0.717	<0.001
Serum calcium (ref = low)	2.885	1.941	3.829	<0.001	1.565	0.746	2.383	<0.001	2.219	1.532	2.906	<0.001	1.738	1.094	2.381	<0.001
Regular physical exercise (ref = never)	−0.788	−2.384	0.807	0.333	−0.562	−1.939	0.816	0.424	−0.810	−1.968	0.349	0.171	−0.177	−1.260	0.905	0.748
Occasionally physical exercise (ref = never)	−2.566	−4.263	−0.869	0.003	−0.720	−2.196	0.756	0.339	−2.468	−3.700	−1.236	<0.001	−0.697	−1.856	0.463	0.239
Dyslipidemia	0.242	−0.935	1.419	0.687	−0.244	−1.256	0.768	0.637	0.695	−0.161	1.551	0.112	0.298	−0.498	1.093	0.463

*HTN, hypertension; BMI, body mass index; %BF, body fat percentage. The classification of low or high in serum calcium level was based on its median value in the overall participants, which was 2.47 mmol/L. Adjusted for sex, age, ethnicity, family history of HTN, BMI categories, serum calcium level, physical exercise and dyslipidemia*.

* *The adjusted covariates excluded BMI due to the high correlation (the Pearson correlation coefficient t = 0.76) between body fat percentage and BMI*.

### The Association of Body Fat Mass and Serum Total Calcium With EBP

Table 3 presented the potential risk factors of EBP. Compared with girls, boys had 2.50 times odds of EBP. Children and adolescents from Hainan Province had 2.87 times odds of EBP than those from Shaanxi Province. Age was found positively associated with EBP. Every 2-year increase in age indicated 1.60 times odds of EBP. Compared with normal weight individuals, lean subjects appeared to have decreased odds of EBP (OR = 0.65, 95% CI: 0.43–0.98) but the overweight/obese ones were more likely to have EBP (OR = 2.67, 95% CI: 1.68–4.25). Children and adolescents with high-level serum calcium were more likely to have EBP, the OR (95% CI) was 1.51 (1.17–1.94).

**Table 3:** The associators of elevated blood pressure in children and adolescents aged 7–18 years in China, 2014.

	**Model 1**				**Model 2**				**Model 3**			
**Covariates**	***OR***	**95%** ***CI*** **of** ***OR***		***P***	***OR***	**95%** ***CI*** **of** ***OR***		***P***	***OR***	**95%** ***CI*** **of** ***OR***		***P***
Boys (ref = girls)	2.406	1.887	3.069	<0.001	2.377	1.858	3.041	<0.001	2.497	1.926	3.239	<0.001
Age (every 2 years increase)	1.497	1.382	1.621	<0.001	1.535	1.414	1.666	<0.001	1.604	1.463	1.760	<0.001
Shaanxi Province (ref = Hainan)	2.239	1.652	3.033	<0.001	2.553	1.867	3.490	<0.001	2.871	1.962	4.202	<0.001
Li ethnicity (ref = Han)	1.105	0.848	1.440	0.458	1.204	0.919	1.578	0.177	0.812	0.601	1.096	0.174
Urban (ref = rural)	1.208	0.939	1.556	0.142	1.124	0.870	1.451	0.101	0.779	0.580	1.045	0.096
Positive Family history of HTN (ref = negative)	1.002	0.773	1.298	0.989	0.955	0.736	1.241	0.733	1.037	0.790	1.362	0.794
Thinness (ref = normal weight)	0.700	0.469	1.046	0.082	0.700	0.469	1.046	0.082	0.648	0.428	0.981	0.040
Overweight/obesity (ref = normal weight)	2.128	1.394	3.248	<0.001	2.128	1.394	3.248	<0.001	2.671	1.679	4.251	<0.001
Body fat percentage (every 5% increase)*	1.114	1.030	1.206	0.007	1.114	1.030	1.206	0.007	1.150	1.054	1.255	0.002
Serum calcium level (ref = low)	1.665	1.300	2.133	<0.001	1.640	1.279	2.103	<0.001	1.508	1.170	1.943	0.001
Regular physical exercise (ref = never)	1.077	0.685	1.693	0.747	1.103	0.700	1.737	0.673	1.189	0.747	1.892	0.464
Occasionally physical exercise (ref = never)	1.354	0.895	2.051	0.152	1.403	0.924	2.129	0.112	1.238	0.810	1.893	0.325
Dyslipidemia (ref = no dyslipidemia)	1.119	0.838	1.495	0.446	1.040	0.775	1.395	0.794	1.107	0.820	1.494	0.506

*Elevated blood pressure was defined as pre-hypertension or hypertension. HTN, hypertension. CI, confidence interval. Model 1: adjusted for age and sex; model 2: further adjusted for BMI (categorial) based on model 1. Model 3: further adjusted for urbanization, study sites, ethnicity, family history of hypertension, serum calcium level, physical exercises, and dyslipidemia based on model 2*.

* *The adjusted covariates for model 2 and model 3 excluded BMI due to its high correlation (the Pearson correlation coefficient = 0.76) with body fat percentage. The classification of low or high in serum calcium level was based on its median value in the overall participants, which was 2.47 mmol/L*.

**Figure 2:**
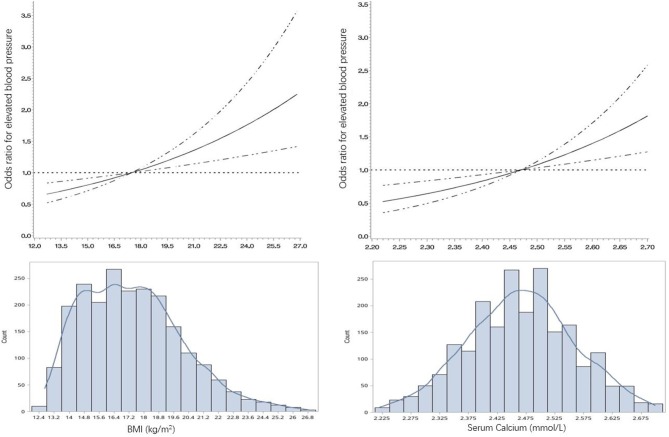
The restricted cubic spline for the association of BMI and serum calcium with OR of elevated blood pressure in children and adolescents aged 7–18 in China, 2014. BMI, body mass index; OR, odds ratio. The lines represent adjusted odds ratios of elevated blood pressure (EBP, including pre-hypertension and hypertension) based on restricted cubic splines for the level of BMI and serum calcium level in a multivariable-Logistic regression model. Adjusted factors for the association between BMI and EBP were age, sex and geographic regions. Adjusted factors for the association between serum calcium and EBP were age, sex, geographic regions, and BMI. The bars represent histograms of BMI and serum calcium distribution among the total participants.

Figure 2 was restricted cubic splines that described the association between BMI, serum calcium and EBP. After adjusting for age, sex and study sites, there was a linear association between BMI and EBP (*P* < 0.001). Similarly, after adjusting for age, sex, study sites and BMI, serum calcium was also observed to have a linear association with EBP (*P* < 0.001).

We further did sub-group analyses within age groups to investigate the associations between body weight and serum total calcium with EBP. However, as limited by the sample size in each age group, only in the 17–18-year group, there was positive association between serum total calcium and EBP (Table 4).

**Table 4:** The association between serum total calcium and body mass index with elevated blood pressure within age groups in children and adolescents aged 7–18 years in China, 2014.

**Age group**	**BMI (ref** **=** **Normal weight)**								**Serum calcium level (ref** **=** **Low)**			
	**Thinness**				**Overweight/obesity**							
	***OR***	**95%** ***CI*** **of** ***OR***		***P***	***OR***	**95%** ***CI***		***P***	***OR***	**95%** ***CI***		***P***
7–10	1.315	0.517	3.342	0.566	1.506	0.555	4.083	0.421	1.796	0.926	3.481	0.083
11–12	0.851	0.334	2.166	0.735	7.520	2.805	20.163	<0.001	1.108	0.585	2.099	0.753
13–14	0.546	0.179	1.671	0.289	3.367	1.043	10.876	0.042	1.892	0.967	3.703	0.063
15–16	0.378	0.134	1.062	0.065	7.993	2.369	26.967	<0.001	1.318	0.729	2.382	0.360
17–18	0.423	0.198	0.905	0.027	0.423	0.198	0.905	0.794	1.633	1.030	2.589	0.037
Overall	0.648	0.428	0.981	0.040	2.671	1.679	4.251	<0.001	1.508	1.170	1.943	0.001

*Elevated blood pressure was defined as pre-hypertension or hypertension. The classification of low or high in serum calcium level was based on its median value in the overall participants, which was 2.47 mmol/L. CI, confidence interval. Independent indicators included in the models included: sex, urbanization, study sites, body mass index categories, and serum total calcium levels*.

## Discussion

This community-based cross-sectional study assessed the prevalence of EBP in Chinese children and adolescents and provided clues in nutritional status and serum calcium as risk factors of high blood pressure, which was, higher serum calcium level was found positively associated with EBP.

The overall prevalence of EBP was 15.42% (8.36% of pre-HTN and 7.06%% of HTN). Geographic and sex difference were observed. Boys, youths living in Hainan Province had higher pre-HTN and HTN prevalence than their counterparts. The disease burden of childhood HTN was varied on geographic distributions. Lu et al. (30) reported a lower prevalence of pre-HTN (6.6%) and HTN (6.0%) in age 7–15 school children in Tianjin Municipality (located in the middle area of China). Yu's study (31) which enrolled children and adolescents aged 12–17 from 31 provinces of China reported a HTN prevalence of 12.4%. A study enrolled 88,974 children and adolescents in Suzhou Province reported prevalence of pre-HTN and HTN as 7.2 and 3.1%, respectively (32). Xi et al. (33) conducted a systematic review which included 11 studies revealed that there was a significant difference in hypertension prevalence across different regions of China, in that youths in North China had higher HTN prevalence than South. In contrary, our study indicated that, subjects living in Hainan Province (located in southern China) had higher EBP prevalence (18.03%) than those living in Shaanxi Province (10.03%, located in northern China). The inconsistency may be attribute to socio-economic disparity in the selected study sites. In Xi's study, the North China areas which included Beijing and Shandong, had better social economic status than Shaanxi Province. One study conducted in Hainan Province among students aged 7–18 in 2009 reported pre-HTN and HTN prevalence of 3.9 and 3.3%, respectively (34), and were lower than what we detected in a more recent time.

The definitions of hypertension in the aforementioned studies were based on blood pressure reference standards for Chinese children and adolescents in 2010 (35), in which stature was not considered and may lead to overestimation of the prevalence (29). Based on the updated blood pressure reference for Chinese children and adolescents initiated in 2017 (29), the overall pre-HTN and HTN prevalence in this study were 10.42 and 10.01%, respectively. For Hainan and Shaanxi Provinces, they were 11.93 and 5.10%, respectively, slightly lower than the results calculated by the updated guidelines of the Forth Report.

As our results suggested that, after adjusting for BMI, urbanization, age and sex, children, and adolescents in Hainan Province had higher risk of EBP than those in Shaanxi Province. The mechanism of this disparity was still unclear. Further studies exploring it from a phenomics prospective will be needed. Boys were more likely to have EBP than girls (OR = 2.50). Sex steroids, such as testosterone may play an important role in influencing blood pressure at puberty (32). The association between physical exercise and EBP was not consistent in previous studies (36–38). This inconsistency was possibly because of different measurement of physical exercise. In this study, physical exercise was classified based on self-reported regular exercise time, more detailed information will be needed to further assess the role of physical exercise influencing BP.

Consistent with previous studies, excess adiposity was found a risk factor of EBP (7, 32). Our results revealed that, body mass index, to some extent as a surrogate of nutritional status in young age, had a linear, positive association with EBP. Overweight/obese children and juveniles had 2.6 times odds of EBP than normal weight counterparts. In contrary, lean individuals had decreased risk of EBP. This finding was also supported by the result of RCS, which indicated a linear association between BMI and EBP. As excess adiposity was a key determinant of EBP during childhood, weight reduction may have important beneficial effect on blood pressure management. In Hu's study (39), individuals who changed from being overweight in childhood to having normal BMI in adolescence had similar mean blood pressures to those who had a normal BMI at both two periods. Since previous studies revealed that BMI had limitations in measuring body composition (40), we used another adiposity index, body fat percentage, to understand the association between body composition and EBP. %BF was also found contributed to childhood EBP (every 5 percent increase in %BF would result in 15% higher risk for EBP). Notably, there was a high prevalence of thinness in Hainan Province (13.80%). Although lean children and adolescents had decreased odds of HTN, there was no reason to encourage people maintain this malnourished status due to its risk for other diseases.

Extracellular calcium level may influence intracellular calcium level and possibly play a role in the pathogenesis of essential HTN (41). The biological mechanisms underlie serum total calcium and blood pressure maybe attribute to a direct effect on vascular by enhanced vascular resistance (42), interaction between serum calcium and other cations (43), alteration in extracellular binding of calcium (44), renal vasoconstriction causing kidney dysfunction (45), and hyperactivity of renin-angiotensin system from hyperparathyroidism (46). Emerging studies have been focused on the role of serum calcium to blood pressure. For children and adolescents, most studies only explored the association between body composition and blood pressure, only a few had investigated potential biomarkers for identifying EBP. The earliest one was among 492 Greek girls aged 12–15, in which higher level of serum total calcium (more than 2.5 mmol/L) had higher systolic blood pressure than those with a lower serum total calcium level (47). In China, Sun's study (48) reported that serum total calcium was found to be positively associated with hypertension among adolescents aged 12–17 in Northeast China. Covering wider age range and different geographic regions, our study revealed that, after adjusting for BMI and other potential confounders, serum total calcium was positively associated with EBP. Furthermore, we applied the restrict cubic splines, by which a linear relationship was observed between serum total calcium value and the OR of EBP. Our findings provided additional impetus of non-traditional risk factors in identifying high risk children and adolescents of EBP in clinical settings.

The limitations of this study should be acknowledged. Firstly, our data were cross-sectional, therefore no conclusions regarding causality can be drawn. Secondly, the present study used single day measurements of blood pressure to identify EBP, which could overestimate the prevalence compared with repeated measurements in different days. However, it is difficult for epidemiological population studies to conduct repeated BP measurements. And based on Zhang's study (14), the risk factors of EBP remained stable during the repeated measurements. We supposed that the association between excess adiposity and serum total calcium with EBP may not be much influenced by the single measurement of blood pressure. Thirdly, we only collected data on serum total calcium but not active/ionized calcium, thus we were not able to compare our results with others in which ionized calcium was tested. Nevertheless, in most clinical settings in China, only serum total calcium is tested for routine health checkups. In addition, test for ionized calcium through blood gas analysis which requires blood sample drawn from artery, is not practical and necessary for children and adolescents for general health examination. Fourthly, the lack of data such as calcium intake and excretion, plasma renin activity, parental demographic characteristics may limit the understanding of risk factors and bias the study results. Lastly, we cannot identify essential hypertensive children due to insufficient diagnostic information, and conclusions from the two study sites will limited their external validity. Nonetheless, with representative community-based sample, vigorous methodology, we investigated the prevalence of high blood pressure in different regions of China and found serum total calcium to be an un-traditional risk factors to identify childhood EBP.

## Conclusions

Body mass index and serum total calcium were found to have linear, positive associations with elevated blood pressure. Serum total calcium could be considered as an untraditional risk factors for high blood pressure along with other body composition indexes in clinical settings to prevent childhood high blood pressure.

## Data Availability Statement

The datasets generated and/or analyzed during the current study are not publicly available due to management rules by the study funder but are available from the corresponding author on reasonable request.

## Ethics Statement

The studies involving human participants were reviewed and approved by the Bioethical Committee of Institute of Basic Medical Sciences, Chinese Academy of Medical Sciences. Written informed consent to participate in this study was provided by the participants' legal guardian/next of kin.

## Author Contributions

GS and HH: conceptualization, methodology, and funding acquisition. HH: software, formal analysis, data curation, writing—original draft preparation, and visualization. GS, HH, and LW: validation, writing—review, and editing. GS, LP, JD, FL, YJ, JM, LW, PJ, and ZH: investigation. GS, JD, and FL: resources. GS and LP: supervision and project administration.

### Conflict of Interest

The authors declare that the research was conducted in the absence of any commercial or financial relationships that could be construed as a potential conflict of interest.
